# Light-Stable Methylammonium-Free Inverted Flexible Perovskite Solar Modules on PET Exceeding 10.5% on a 15.7 cm^2^ Active Area

**DOI:** 10.1021/acsami.1c05506

**Published:** 2021-06-16

**Authors:** Luigi Angelo Castriotta, Rosinda Fuentes Pineda, Vivek Babu, Pierpaolo Spinelli, Babak Taheri, Fabio Matteocci, Francesca Brunetti, Konrad Wojciechowski, Aldo Di Carlo

**Affiliations:** † Centre for Hybrid and Organic Solar Energy (CHOSE), Department of Electronic Engineering, University of Rome Tor Vergata, Rome 00133, Italy; ‡ Saule Technologies, Wroclaw 54-427, Poland; § Saule Research Institute, Wroclaw 54-427, Poland; ∥ Institute for Structure of the Matter—National Research Council (ISM−CNR), via del Fosso del Cavaliere 100, Rome 00133, Italy

**Keywords:** perovskite solar modules (PSMs), fabrication, flexible perovskite solar modules, light stability

## Abstract

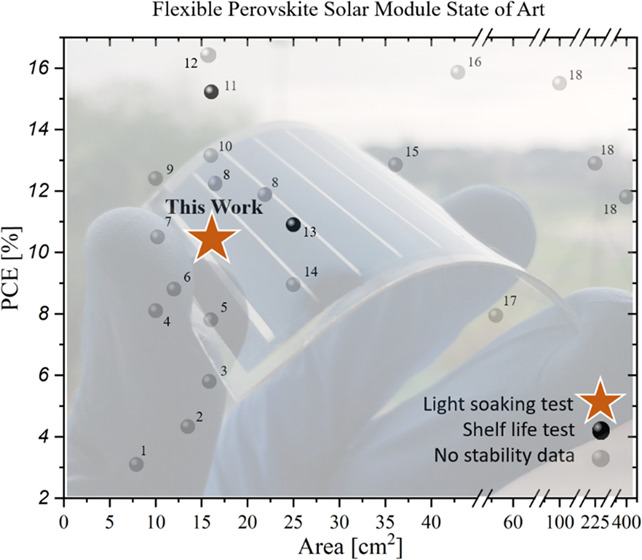

Perovskite solar modules (PSMs) have been attracting the photovoltaic market, owing to low manufacturing costs and process versatility. The employment of flexible substrates gives the chance to explore new applications and further increase the fabrication throughput. However, the present state-of-the-art of flexible perovskite solar modules (FPSMs) does not show any data on light-soaking stability, revealing that the scientific community is still far from the potential marketing of the product. During this work, we demonstrate, for the first time, an outstanding light stability of FPSMs over 1000 h considering the recovering time (*T*_80_ = 730 h), exhibiting a power conversion efficiency (PCE) of 10.51% over a 15.7 cm^2^ active area obtained with scalable processes by exploiting blade deposition of a transporting layer and a stable double-cation perovskite (cesium and formamidinium, CsFA) absorber.

## Introduction

The strong interest exhibited by the international scientific community to the perovskite solar cells^1^ (PSCs) permitted a tremendous improvement in the performance of this photovoltaic (PV) technology in recent years, reaching a power conversion efficiency (PCE) of 25.5%^2^ in 2020, thus closely approaching crystalline silicon solar cells. The success of halide perovskite is mainly due to its high charge-carrier diffusion lengths, a high optical absorption coefficient, low exciton binding energy, and facile tuning of the band gap by simply playing with the precursor components.^3−5^ Moreover, the possibility of performing a low-temperature deposition process of the entire PSC structure permitted extending the manufacturing to flexible polymeric substrates.^6−8^ Flexible perovskite solar cells (FPSCs) provided access to new applications not suitable for traditional photovoltaic technologies, such as adaptive photovoltaics, transportable electronic chargers, wearable electronic devices, sensors for the Internet of Things (IoT), etc., which attracted considerable attention from both the scientific and industrial communities.^9,10^ The FPSC development started back in 2013, where Kumar et al. used low-temperature annealing (below 100 °C) to prepare zinc oxide (ZnO) nanorods as an electron transport layer (ETL), showing the first-ever FPSCs with a PCE of 2.62%.^11^ Among others, Shin et al. fabricated a nanocrystalline Zn_2_SO_4_ film with improved optical transmittance to replace standard titania (TiO_2_) for ETL in FPSCs, reaching an efficiency of 15.3%.^12^ Recently, Chung et al. were able to reach 20.7% efficiency using Zn_2_SnO_4_ as a porous planar ETL.^13^ Most of these studies and achievements have been performed on a small-area device (with active area equal or lower to 0.1 cm^2^),^14−17^ while fewer studies are available on large-area FPSCs,^18,19^ and even less on flexible perovskite solar modules (FPSMs, see Table 1): as far as we know, only 18 studies show results of perovskite modules on flexible substrates, and not one of them focuses on light stability of such modules; only Bu et al.^20^ and Hu et al.^21^ demonstrated a shelf-life stability of 1000 and 550 h, respectively.

**Table 1:** List of Flexible Perovskite Solar Module Publications Updated up to May 25, 2021^a^

progressive number	device structure	perovskite deposition technique	area [cm^2^]	PCE [%]	stability data	reference number
1	PET/ITO/TiO_2_/m-TiO_2_/CH_3_NH_3_PbI_3–*x*_Cl*_x_*/Spiro-MeOTAD/Au	spin-coating	7.92	3.1	no	(22)
2	PEN/ITO/TiO_2_-np/MAPI/Spiro-MeOTAD/Au	spin-coating	13.5	4.33	no	(23)
3	PET/ITO/SnO_2_-np/CH_3_NH_3_PbI_3–_*_x_*Cl*_x_*/Spiro-MeOTAD/Ag	spin-coating	15.84	5.8	no	(24)
4	PEN/ITO/MFGO/MAPI/PCBM/BCP/Ag	spin-coating	10	8.1	no	(25)
5	PEN/ITO/C60/MAPI/Spiro-MeOTAD/MoO_3_/Au	thermal evaporation (PbI_2_) + spin-coating (MAI)	16	7.8	no	(26)
6	PET/ITO/SnO_2_/m-TiO_2_/MAPI/Spiro-MeOTAD/Au	spin-coating	12	8.8	no	(27)
7	Flexible-Substrate/AZO/LiF/C60/ MAPI/Spiro-MeOTAD/Au	thermal evaporation (PbI_2_) + spin-coating (MAI)	10.2	10.5	no	(28)
8	PET/ITO/SnO_2_/ Cs_0.08_FA_0.78_MA_0.16_Pb (I_0.84_Br_0.16_)_3_/Spiro-MeOTAD/Au	spin-coating	16.84	12.0	no	(29)
			21.84	11.7		
9	PET/ITO/SnO_2_/K_0.03_Cs_0.05_(FA_0.85_MA_0.15_)_0.92_Pb(I_0.85_Br_0.15_)_3_/Spiro-MeOTAD/Au	spin-coating	10	12.4	no	(30)
10	Flexible-Substrate/ITO/2T-NATA/MAPI/C60/BCP/Ag	thermal evaporation	16	13.15	no	(31)
11	PET/ITO/SnO_2_-NCs/KOH/Cs_0.05_(FA_0.85_MA_0.15_)_0.95_Pb (I_0.85_Br_0.15_)_3_/Spiro-MeOTAD/Au	spin-coating	16.07	15.22	shelf life	(20)
12	PET/hc-PEDOT-PSS/HI-NiO*_x_*/perovskite/PCBM/BCP/Ag	R2R printing	15	16.5	no	(32)
13	PET/PEDOT:PSS:CSE/Perovskite/ PCBM/Ag	spin-coating	25	10.9	shelf life	(21)
14	PET/ITO/PEDOT:PSS/Cs_0.1_FA_0.7_MA_0.2_PbBr_0.2_I_2.8_/EVA/PCBM/BCP/Ag	spin-coating	25	8.95	no	(33)
15	PET/ITO/NiO-np/PMMA/FA_0.79_MA_0.16_PbBr_0.51_I_2.49_/ PCBM/BCP/Ag	spin-coating	36.1	12.85	no	(34)
16	MgF_2_/Willow glass/ITO/PTAA/MAPI-NH_4_Cl/C60/BCP/Cu	blade-coating	42.9	15.86	no	(35)
17	PDMS/PEDOT:PSS/FA_0.83_MA_0.17_PbBr_0.51_I_2.49_/PCBM/PEI/PEDOT:PSS/PDMS	spin-coating	56	7.95	no	(36)
18	PEN/ITO/SnO_2_/Zn_2_SnO_4_/ (FAPbI_3_)_0.95_(MAPbBr_3_)_0.05_/2D Perovskite/Spiro-MeOTAD/Au	spin-coating	100, 225, 400	15.5, 12.9, 11.8	no	(13)
this work	PET/ITO/PTAA/Cs_0.17_FA_0.85_Pb(I_0.9_Br_0.1_)_3_/C60/BCP/Ag	N_2_ assisted blade-coating	15.7	10.51	light soaking	this work

a Keywords used on Google Scholar: flexible perovskite solar module.

The solution chemistry and process conditions used for lab-scaling techniques, such as spin-coating, cannot be easily transferred to scalable deposition techniques: the thinning and smoothing of wet-solution films in the spin-coating process are dependent on the constant centrifugal force of spinning, which is difficult to reproduce in scalable deposition processes.^37^ Thus, up-scaled deposition techniques are necessary to develop suitable demonstrators that permit transferring the knowledge from a lab scale to a preindustrial dimension.^38,39^ In this context, the blade-coating deposition method is a suitable scalable technique. Compared with spin-coating, the scalability of the process is favorable for several reasons: the spreading of the solution is not affected by the size of the substrate.^40^ Moreover, the ink waste is strongly minimized compared to spin-coating.^41^ Stability of perovskite devices is becoming mandatory and extremely useful when exploiting this technology. In 2020, a consensus among university and research centers in this field was reached on standardizing stability measurements for perovskite solar cells, showing the importance of making stability measurements;^42^ several factors impact the stability of PSCs^43^ and, among them, the composition of the perovskite material could master both intrinsic and extrinsic stability.^44^ In the consensus statement provided by Khenkin et al., stress tests specific to modules are not discussed. A detailed ISOS standard stress test might be included in the future specifically for flexible modules, including potential-induced degradation, bypass diode stability, mechanical stability, hail tests, and special consideration for space applications. Until now, perovskite module stress tests indeed are likely only included from the existing IEC 61215 standard.^45^ In conventional PSCs, the use of methylammonium (MA), as a volatile compound, facilitates degradation when exposed at a relatively high temperature (above 85 °C).^46,47^ Various studies have shown that methylammonium-based perovskites are unstable, with film degradation due to degassing.^46,48−52^ Recently, many researchers proved that double-cation perovskite, using cesium and formamidinium (CsFA),^53,54^ shows better intrinsic stability on small-area cells (up to 0.1 cm^2^ area), even though only a few tests have been done so far on large-area devices, including modules, with promising results on conventional architecture and a rigid substrate.^55^ A focus on module stability is needed to overcome possible degradation effects at the interconnections, mainly when putting a contact metal with a perovskite layer.^56−60^ The choice of the perovskite material, avoiding MA containing perovskite, is a game changer if the focus on longer lifetime is tested, enabling longer stability from a material to a device level.^44,52^

Herein, for the first time, we present an outstanding light-stable methylammonium-free inverted perovskite solar module on a poly(ethylene terephthalate) (PET) flexible substrate, showing stability under continuous light soaking with a *T*_80′_ of 730 h. Moreover, the best module showed a *T*_80″_ of 1560 h after a recovery in the dark of 96 h, generating 164.8 mW with a PCE of 10.51% on a 15.7 cm^2^ active area. Our approach aims at innovating, as it avoids any use of lab-scaling techniques, including the optimization of poly[bis(4-phenyl)(2,4,6-trimethylphenyl)amine] (PTAA) and cesium-formamidinium (CsFA) perovskite layer depositions using blade-coating and N_2_-assisted blade-coating, respectively. PTAA and perovskite deposition by blade-coating are proposed as the main ways to boost the light stability by limiting the charge losses occurring at the PTAA/perovskite layer. To this end, a complete study, first on cells’ matrix and then on module size, has been carried out by varying solution concentrations, blade speeds, and height parameters.

## Results and Discussion

**Figure 1:**
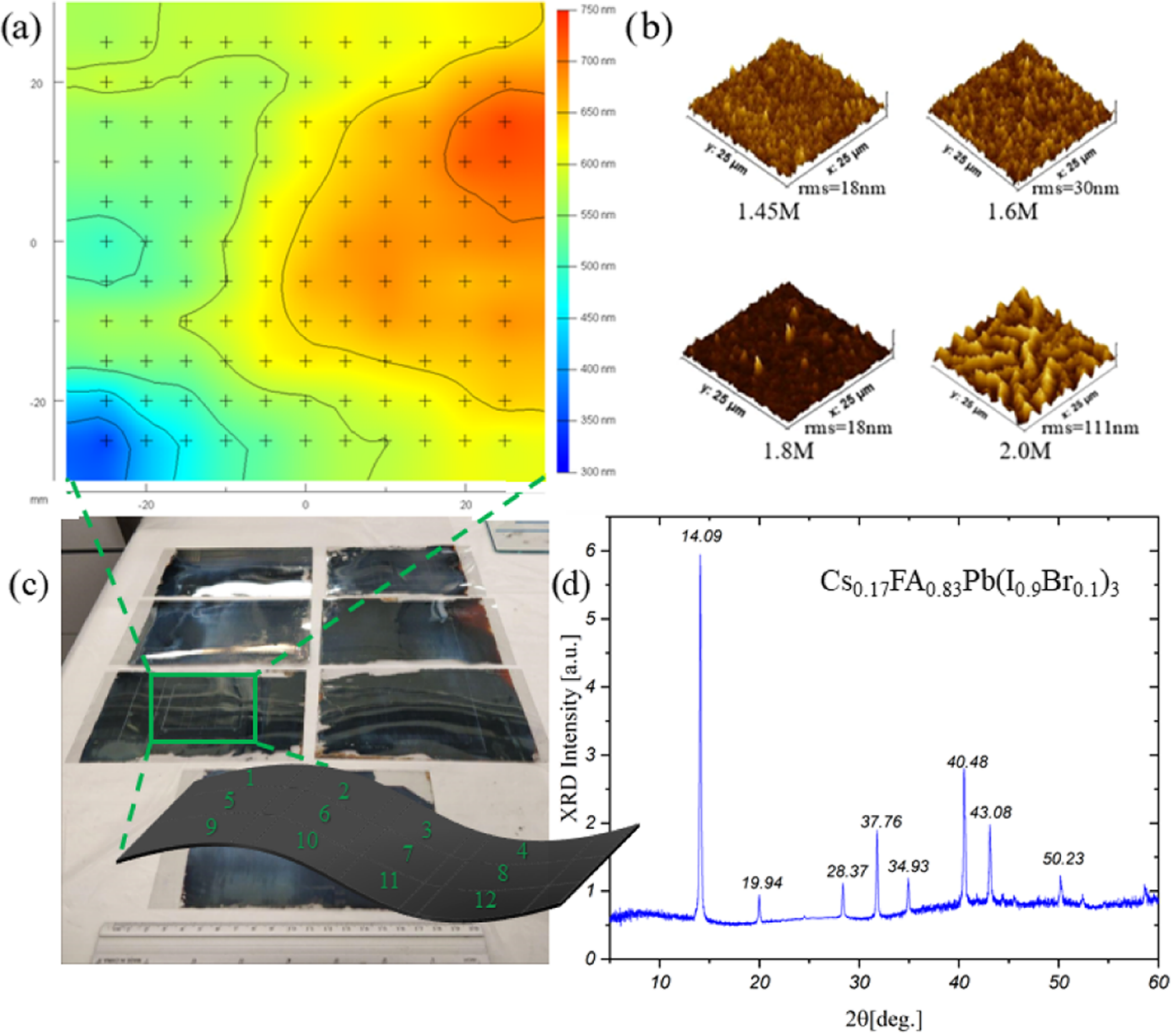
(a) Ellipsometry image of the perovskite film thickness study at a concentration of 1.8 M. The average thickness was found to be 571.3 ± 54.9 nm. (b) Atomic force microscopy (AFM) images of perovskite roughness at concentrations of 1.45, 1.6, 1.8, and 2.0 M. (c) Images of the perovskite film with a sketch of the 12 device pattern and (d) X-ray diffraction (XRD) spectra of the 1.8 M CsFA perovskite.

The aim of this work is to optimize PTAA and perovskite deposition using an up-scalable printing technique. In this frame, we developed a deposition strategy by varying the solvent system, concentration, and blade parameters for both the PTAA and perovskite layer. We used the same PET/indium-doped tin oxide (ITO) substrate size of 5 × 7 cm^2^ to realize a matrix of 12 devices of 0.616 cm^2^ and a module active area of 15.7 cm^2^, as shown in Figure 1c. We changed the solvent system to both the PTAA and perovskite layer; the PTAA layer was optimized using anisole instead of toluene mainly for safety reasons: we deposited the solution in air in a clean-room environment and wanted to limit any kind of dangerous solvents in the atmosphere. Three concentrations were studied: 2, 5, and 10 mg/mL. The blade height and speed were kept fixed at 100 μm and 5 mm/s, respectively. The best candidate for the perovskite deposition was determined based on the thickness and roughness of the layer: 5 mg/mL was the best concentration and agreed with the literature findings for this kind of a layer and a structure, with an average thickness value of ∼5.7 ± 0.5 nm. The summary of the PTAA parameters optimized and the ellipsometry map of the PTAA at a concentration of 5 mg/mL are shown in Table S1 and Figure S1, respectively.

The perovskite layer was optimized starting from a standard double-cation recipe found in the literature.^61^ We changed the solvent system from a standard *N*,*N*-dimethylformamide/dimethyl sulfoxide (DMF/DMSO) 4:1 volume ratio used mainly for the spin-coating process to *N*-methyl-2-pyrrolidone (NMP)/DMF 95:5 already optimized for the blade-coating process in a previous report.^62^ The study on the concentration was carried out using 1.45, 1.6, 1.8, and 2 M concentrations. Perovskite deposition was performed using N_2_-assisted blade-coating in a N_2_-filled glovebox environment. Perovskite film pictures are shown in Figure 1c, together with a scheme representation of the matrix used for a small-area device (12 devices by a 0.616 cm^2^ active area). For N_2_-assisted blade parameters, we fixed N_2_ pressure at 2 bar and varied the blade height and speed. Table 2 shows the principal perovskite parameters used together with thickness variation. Figure 1b shows the average roughness of the different perovskite concentrations analyzed. For all types of perovskite concentrations, a photoluminescence (PL) peak was found at ∼772 nm, as shown in Figure S2.

**Table 2:** Summary of the Perovskite Parameters Optimized and Thickness Values Calculated from Four Different Films from the Same Matrix

concentration [M]	blade height [μm]	blade speed [mm/s]	thickness [nm]	roughness, rms [nm]
1.45	70	1.5	∼262	∼18
	100	1.5	∼320	
	70	3	∼180	
	100	3	∼273	
1.6	70	1.5	∼254	∼30
	100	1.5	∼304	
	70	3	∼220	
	100	3	∼299	
1.8	70	1.5	∼554	∼18
	100	1.5	∼628	
	70	3	∼405	
	100	**3**	∼464	
2.0	70	1.5	∼694	∼111
	100	1.5	∼721	
	70	3	∼778	
	100	3	∼826	

An optimized concentration of 1.8 M was found to be the best concentration for device fabrication, showing thickness data close to literature ideal values for charge extraction in perovskite solar cell technology.^63^ A perovskite film at a 1.8 M concentration was further studied with ellipsometry, as shown in Figure 1a: the average thickness found along the module was 571.3 ± 54.9 nm, also proven by scanning electron microscopy (SEM) cross section image (Figure S4). As a step forward to our fabrication process, we finalized the device using the matrix device scheme (Figure 1c) at a perovskite concentration of 1.8 M. To be able to focus fully on the perovskite thin-film quality and have good reproducibility of the device results, C60/bathocuproine (BCP) and Ag processed by thermal evaporation were selected to ascertain a good layer morphology and provide high and uniform coverage of the perovskite surface. In this way, significantly improved reproducibility could be obtained, which helped in the optimization work of perovskite thin-film deposition trials. The device architecture used was as follows: PET/ITO/PTAA/Cs_0.17_FA_0.83_Pb(_0.9_Br_0.1_)_3_/C60/BCP/Ag.

**Figure 2:**
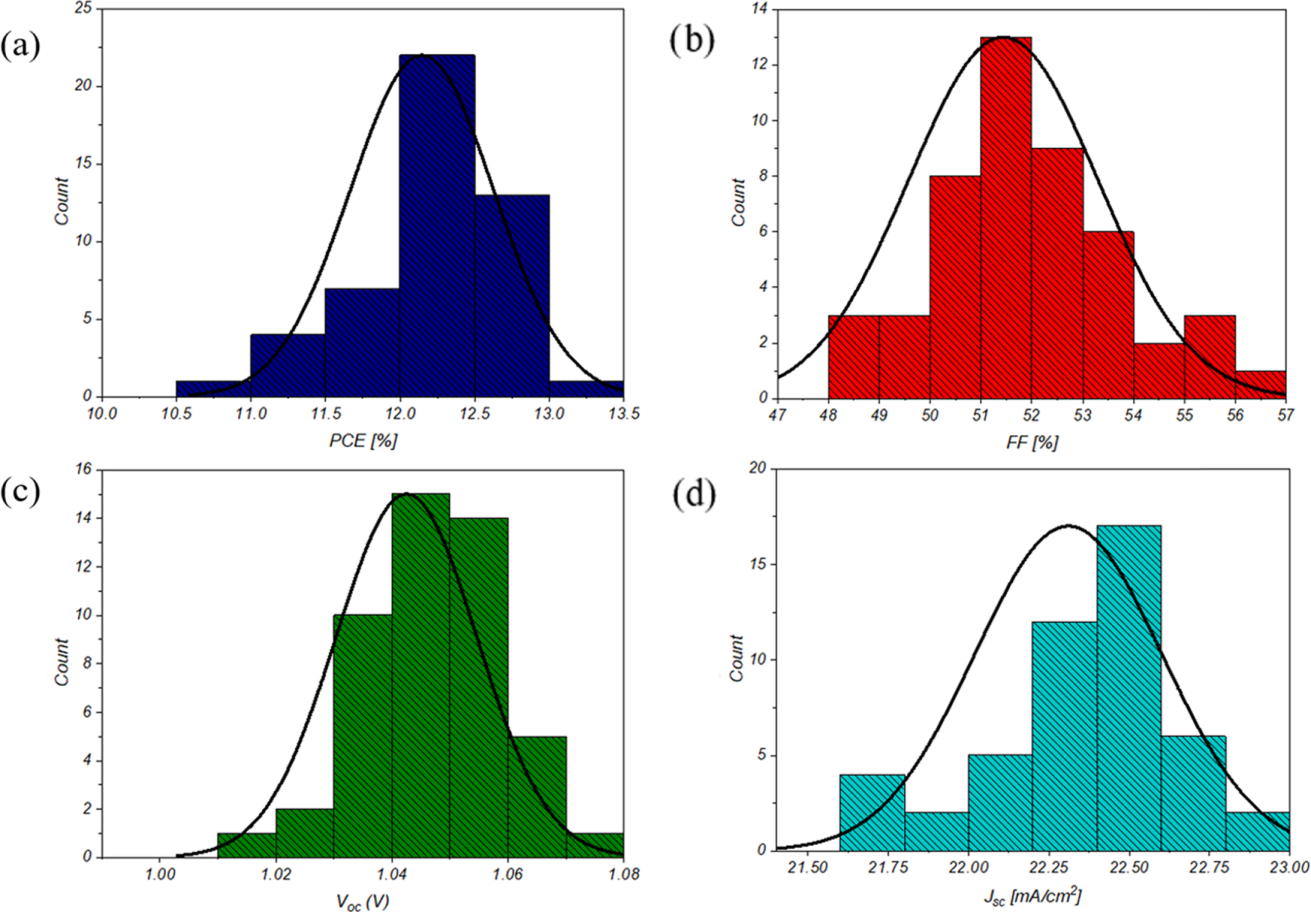
Statistics of PCE (a), FF (b), *V*_oc_ (c), and *J*_sc_ (d) obtained on 48 different devices, with an active area of 0.616 cm^2^ each, fabricated on four matrix substrates with a 1.8 M perovskite concentration.

**Figure 3:**
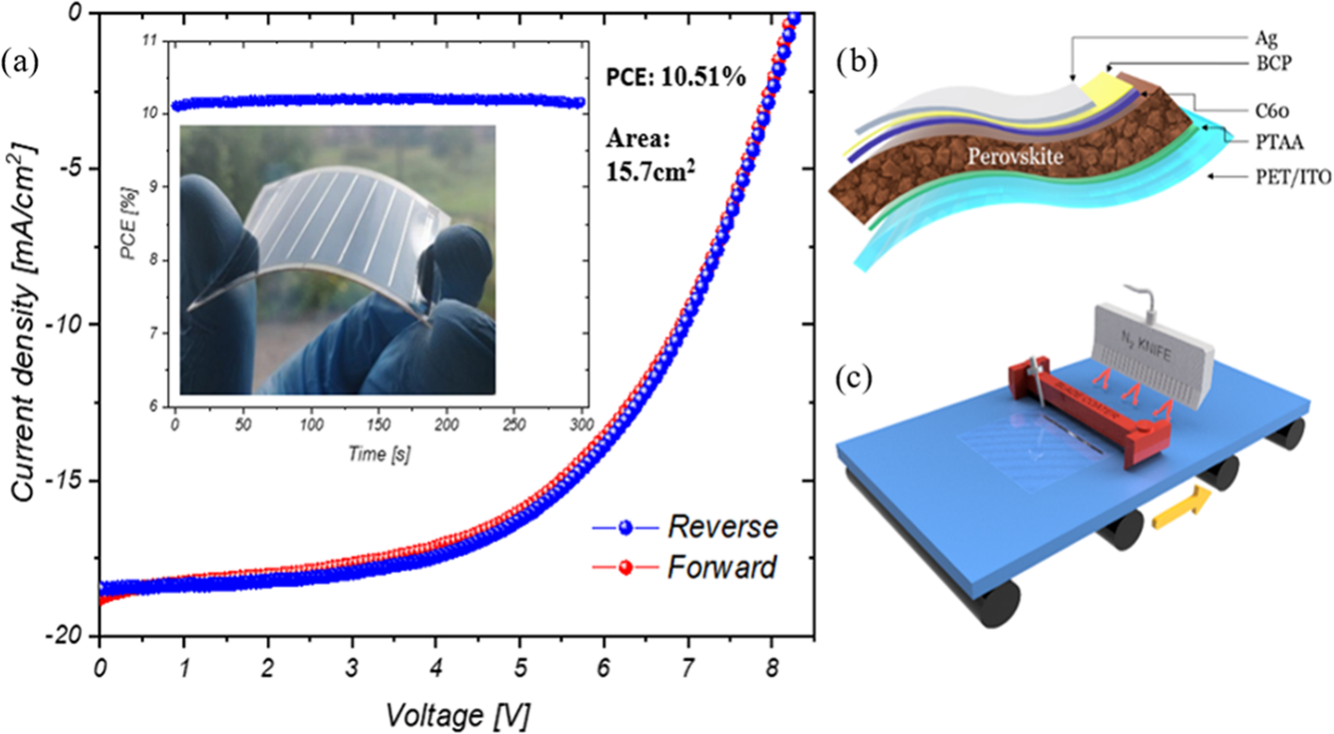
(a) *J–V* curve and 5 min stabilized efficiency (maximum power point (MPP) tracking) of the best module with an active area of 15.7 cm^2^. (b) Structure of the perovskite solar module used. (c) Scheme of the perovskite deposition technique used.

Four different matrix substrates have been fabricated by varying the blade height (70 and 100 μm) and speed (1.5 and 3 mm/s) using the same perovskite concentration (1.8 M). Figure 2 shows the statistical distribution of the main PV parameters for the fabricated cells. In general, we did not identify a significant variation by changing the blade height and speed. At the same time, we notice that it is fundamental, when working on the PET substrate, to make sure that the substrate is well attached to the blade working stage; every tiny variation, very common and almost impossible to avoid when working on big substrates, might be relevant and it could create a thickness mismatch for the upcoming material depositions. The champion device has a PCE of 13.02%, FF of 54.06%, *V*_oc_ of 1.06 V, and *J*_sc_ of 22.69 mA/cm^2^ (for details, see Figure S3 and Table S2) and was obtained with a blade height and speed of 70 μm and 1.5 mm/s, respectively. Based on these results, the scaling-up studies performed in this work have been obtained with these parameters for the blade height and speed for both PTAA and perovskite layers. The *JV* characteristics of the best module including the stabilized efficiency are shown in Figure 3a and in Table 3. A schematic of the device stack and the technique used are shown in Figure 3b,c, respectively.

**Table 3:** Summary of the *J*–*V* Results Obtained on Modules Fabricated on Best and Average Data Using Standard Deviation Based on Four Different Modules

scan		efficiency [%]	fill factor [%]	*V*_oc_ [V]	*J*_sc_ [mA/cm^2^]	*P* [mW]
reverse	best	10.51	55.03	8.26	18.48	164.8
	average	9.83 ± 0.75	53.67 ± 6.34	8.12 ± 0.15	16.77 ± 2.27	152.7 ± 9.63
forward	best	10.32	53.67	8.21	18.74	161.7
	average	9.71 ± 0.51	51.90 ± 2.51	8.08 ± 0.14	16.69 ± 2.11	153.47 ± 8.81

A champion module with eight series-connected cells with a total active area of 15.7 cm^2^ exhibited a PCE of 10.51%. Average data based on four modules fabricated using same parameters showed a PCE of 9.83 ± 0.75%.

**Figure 4:**
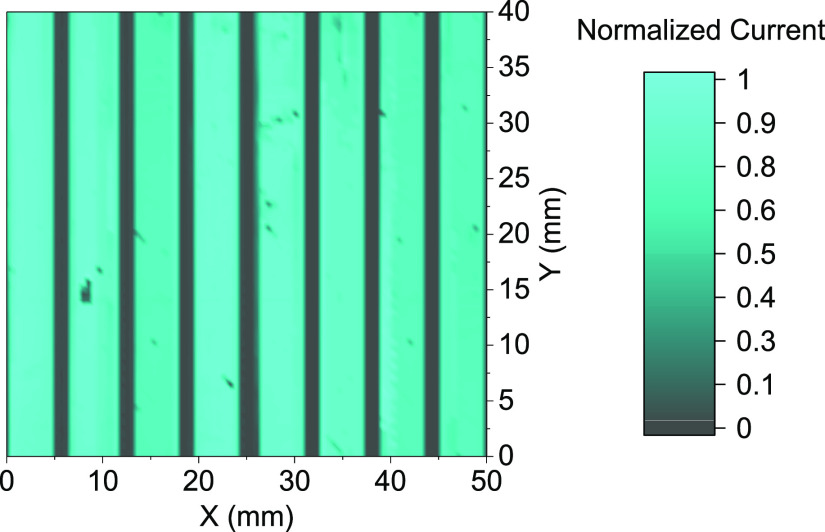
LBIC map of the fabricated modules. The area was scanned following a standard procedure.^41,55^

The homogeneity of the perovskite solar modules was assessed using light beam-induced current (LBIC), an effective characterization technique for mapping large-area devices and checking device uniformity.^41,64^Figure 4 shows the LBIC map for the best module fabricated. The map reports normalized current, which is obtained by scanning the entire area of the module, including the interconnections done by laser patterning. Inhomogeneities like pinholes are only a few in the module, and their effect on device performances is limited.

**Figure 5:**
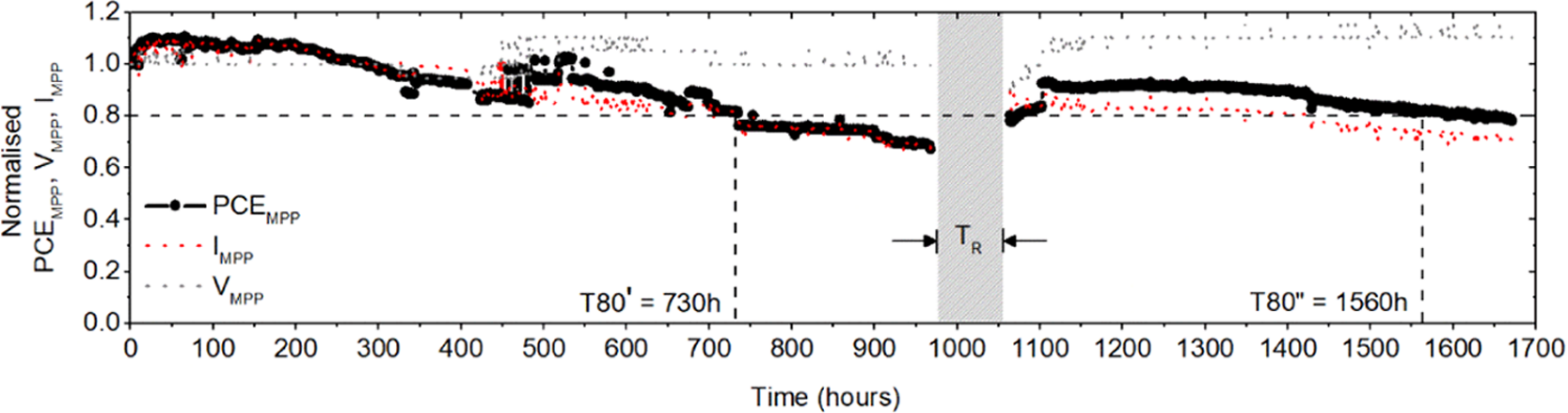
Light-soaking test of the best module fabricated showing the temporal profiles of normalized PCE_MPP_ (black circle curve), *V*_MPP_ (gray dotted curve), and *I*_MPP_ (red dotted curve) values. The stability test was performed in an air environment for 1670 h under 1 sun as a light condition at MPP, with 395–780 nm λ range. The initial light-soaking test was performed for 1000 h followed from the second test after a recovery time (*T*_R_) of 96 h where the module was stored in the dark.

Light-stability experiments on perovskite modules were conducted to thoroughly explain the degradation effects: we decided to perform such studies on modules because we wanted to concentrate on the real degradation effects that could occur in a future application out of a research lab. As a result, the fabricated module was subjected to a light-soaking test in an air environment following the ISOS-L-1 light-stability protocol^42^ defined for PSC devices. The stability test was performed polarizing the module at a maximum power point (MPP) and then tracked using an MPPT algorithm for 1000 h. Then, the MPPT was stopped for a recovery time (*T*_R_) where the module was stored in air for 96 h in dark conditions.^65^ Finally, the device was again measured under MPPT for a total duration of 1670 h. The results are shown in Figure 5, where the temporal profiles of the normalized PCE_MPP_, *V*_MPP_, and *I*_MPP_ are reported. The main parameter evaluated during the light-soaking test is the *T*_80_ parameter defined as the time where 80% of the initial performance is retained from the module. An impressive *T*_80′_ of 730 h was acquired during the initial light soaking. To our knowledge, this result represents the highest value from the flexible PSM module reported in the literature. The sample did not show irreversible degradation: after storage in the dark for 96 h, the module was again light-soaked at MPPT, partially recovering its initial efficiency (up to 95% of the initial efficiency). The recovery in the dark mainly improved the current at MPPT: this behavior might be related to the trap filling after the reillumination, as also discussed by Khenkin et al.^66^ A *T*_80″_ equal to 1560 h was measured during the second light-soaking test demonstrating reversible degradation of the initial photovoltaic performance.

This result shows that continuous light soaking is very severe for the perovskite modules and does not quite well represent the outdoor operative conditions where the light/dark alternation can reduce the module degradation, as also discussed by Khenkin et al., which showed that PSC degradation occurs in a variety of reversible and irreversible processes under real-world operating conditions.^65,66^ These results showed the importance of using light/dark cycling in PSC stability.

## Experimental Section

### Materials

The PET substrates coated with ITO (sheet resistance of 60 Ω/sq) were obtained from Kintec Company and PTAA was obtained from Osilla. The remaining products were obtained from Sigma-Aldrich and used without further purification.

### Perovskite Solutions

Cs_0.17_FA_0.83_Pb(I_0.9_Br_0.1_)_3_ with a concentration of 1.45 M was prepared as follows: 64 mg of CsI, 79.8 mg of PbBr_2_, 207 mg of FAI, and 568.2 mg of PbI_2_ were dissolved in one pot in 1 mL of a 95:5 NMP/DMF volume ratio. Different concentrations of 1.6, 1.8, and 2.0 M were prepared with a simple molar ratio comparison from a 1.45 M starting concentration.

### Device Fabrication

The following architecture was used to make planar heterojunction PSCs and PSMs: PET/ITO/PTAA/Cs_0.17_FA_0.83_Pb(I_0.9_Br_0.1_)_3_/C60/BCP/Ag. Flexible solar cells with an active area of 0.616 cm^2^ and perovskite solar modules (PSMs) with an active area of 15.7 cm^2^ (starting from a substrate size of 130 × 180 mm^2^) were realized on indium-doped tin oxide (ITO) conductive PET. The ITO on a PET substrate (P1) was patterned using the laser from Rofinpowerline E25 (λ = 1064 nm) for both small-area devices and mini modules for interconnects. The substrates were sonicated in deionized (DI) water and isopropanol. A 2 min oxygen plasma treatment was performed prior to layer processing. A PTAA solution in anisole at different concentrations (1, 5, and 10 mg/mL) was blade-coated in a clean-room environment using 100 μm height and 5 mm/s speed, followed by annealing at 100 °C for 10 min. Subsequently, the samples were exposed to UV light for 10 min and then transferred into a nitrogen-filled glovebox for perovskite layer deposition. A perovskite layer was deposited by N_2_-assisted blade-coating with a fixed N_2_ pressure of 2 bar, using different blade-coating parameters summarized in Table 3. The intermediate phase formed after the drying process was completely crystallized by transferring the samples to an oven at 100 °C for 45 min. On top of the perovskite layer, 30 nm of C60 as ETL and 8 nm of BCP as a buffer layer were thermally evaporated at a vacuum pressure of ≈10 to 6 mbar. To form interconnects in the module, all of the layers except ITO (P2) were laser patterned using the same laser type for P1. The laser ablation was performed using the parameters optimized in Saule Technologies. Finally, as the back-contact, 100 nm of a Ag electrode was deposited on top of the layers by thermal evaporation at ≈10 to 6 mbar by a shadow mask (P3). It is important to carefully align the shadow mask with P1 and P2 lines on the module before Ag evaporation (see Figure S4 for details). The modules fabricated by P1, P2, and P3 patterning consist of eight cells connected in series. All device characterizations performed are fully described in the Supporting Information.

## Conclusions

A large-area flexible perovskite solar module has been demonstrated with a fully scalable deposition technique: the results show the optimization of PTTA and perovskite layer deposition by blade-coating, with the final fabrication of a flexible perovskite module with a PCE of 10.51% over 15.7 cm^2^, showing outstanding light stability of FPSM with a *T*_80′_ of 730 h and a recovery efficiency in the dark showing a *T*_80″_ of 1560 h, the most stable to the best of our knowledge in the literature reported so far. The use of scaling-up techniques, such as a blade-coating process, in the field of perovskite opens a feasible path to further increase the stability of flexible modules while keeping this technology repeatable, cheaper, and suitable for flexible solar panel technology. We believe that a light/dark cycle test, such as ISOS-LC standards, could be integrated to understand and study the recovery process of the devices under dark conditions, simulating the night and day shifts.

## Supporting Information Available

Methods describing IV measurements, PL characterization, external quantum efficiency (EQE), AFM, ellipsometry, LBIC, and light-soaking measurements; summary of the PTAA parameters optimized, PTAA ellipsometry map, PL, *J*–*V* curves, EQE spectra of the best small-area solar cell device optimized, and the SEM cross section of the stack (PDF)
Video illustration of the blade coating perovskite deposition (MP4)
