# Supplementary material for: Light-Stable Methylammonium-Free Inverted Flexible Perovskite Solar Modules on PET Exceeding 10.5% on a 15.7 cm2 Active Area
Source: ACS Appl Mater Interfaces. 2021 Jun 16;13(25):29576–84. doi: 10.1021/acsami.1c05506 (PMC8289250; doi:10.1021/acsami.1c05506)
Supplement: Supplementary file 1 — am1c05506_si_001.pdf [file am1c05506_si_001.pdf]

Supporting Information for

Light Stable Methylammonium-Free Inverted Flexible Perovskite Solar Module on PET exceeding 10.5% on 15.7cm^2 active area

*Luigi Angelo Castriotta[1], Rosinda Fuentes Pineda[2], Vivek Babu[1,2], Pierpaolo Spinelli[2], Babak Taheri[1], Fabio Matteocci[1], Francesca Brunetti[1], Konrad Wojciechowski[2,3], Aldo Di Carlo[1,4] **

[1] Centre for Hybrid and Organic Solar Energy (CHOSE), Department of Electronic Engineering, University of Rome Tor Vergata, Rome 00133, Italy

[2] Saule Technologies, Wroclaw 54-427, Poland

[3] Saule Research Institute, Wroclaw 54-427, Poland

[4] Institute for Structure of the Matter – National Research Council (ISM –CNR), via del Fosso del Cavaliere 100, Rome 00133, Italy

***aldo.dicarlo@uniroma2.it**

ADDITIONAL DATA

I-V Measurement. I−V characteristics are measured with a class A sun simulator (ABET) under AM 1.5G 1 sun illumination condition. The AM1.5G condition is obtained by using an optical filter. The sun simulator was calibrated using a Si reference cell (RR-226-O, RERA Solutions). An active area of 0.616 cm^2 was determined by a metal mask. Active area of modules were defined by laser etching, by multiplying active area width and height by number of cells, with total active area of 15.7cm^2. The J-V curves were scanned with the rate of 20 and 300 mV s^{-1} for cells and modules respectively without any preconditioning. The PV parameters are extracted from reverse scan direction (from Voc to Isc). The MPPT protocol realizes the first I−V scan in forward direction to find the MPP condition; then, the I−V tracking maintains the device under MPP applying a small perturbation of both V_{MPP} and J_{MPP} to obtain the dynamic MPP value for 300 s.

PL Characterization. Steadystate PL and electroluminescence measurements were performed with a commercial apparatus (Arkeo, Cicci Research s.r.l.) composed by a charge-coupled device spectrometer. The substrates were excited by a green (532 nm) laser at 45° of incidence with a circular spot diameter of 1 mm. The optical coupling system is composed by a lens condenser attached to a multimode optical fiber bundle. After waiting an integration time of 100 ms, the PL signal is acquired for each sample.

External Quantum Efficiency. The EQE was measured using Bentham PVE300 photovoltaic characterization system and the control software BenWin+.

Atomic Force Microscopy. The AFM images were obtained using a Park Systems, Model XE7 in noncontact mode, and scanning over a range of 25 μm by 25 μm at a resolution of 128 × 128 data points. The surface roughness was measured as the root mean-squared roughness over the scanning area.

Ellipsometer Measurement. The thicknesses of the PTAA and perovskite layers were measured by spectroscopic ellipsometry. Measurements were performed in a Semilab SE2000 ellipsometer, at a 70-degree angle for a wavelength range 245-967nm. The dielectric constants of PTAA were modelled by a Tauc-Lorentz (TL) and a Gaussian function. The thickness was measured in different points with a spacing of 0.5 cm from each other. The color maps show the thickness variation across the sample. For PTAA, an average thickness of 5.7nm was measured, with a standard deviation of 0.5nm.

LBIC Measurement. Spatially resolved photocurrent maps were measured following a procedure elaborated in our previous work[1].

Light Soaking Measurement. Stability tests were performed in air environment with an Arkeo-multichannel (Cicci Research company) based on 32 fully independent Source Meter Unit (+/- 10V @ +/-250 mA) and an ARKEO Light soaker (VIS version) with low mismatch LED based system (400-750nm). A standard Perturb & Observe tracking algorithm was selected with a JV scan every 30 minutes. Modules were encapsulated prior to the test.

Table S1. Summary of the PTAA parameters optimized.

Concentration [mg/ml]	*Solvent*	*Blade Height [μm]*	*Blade Speed [mm/s]*	*Thickness [nm]*	*Roughness, rms [nm]*
1	Anisole	100	5	undetectable	~7
5	Anisole	100	5	~6	~5
10	Anisole	100	5	~25	~6

Figure S1. Ellipsometry map of PET/ITO/PTAA; 5mg/ml concentration used. Average thickness calculated with Standard deviation: 5.7±0.5nm. Tauc-Lorentz and Gaussian model used.

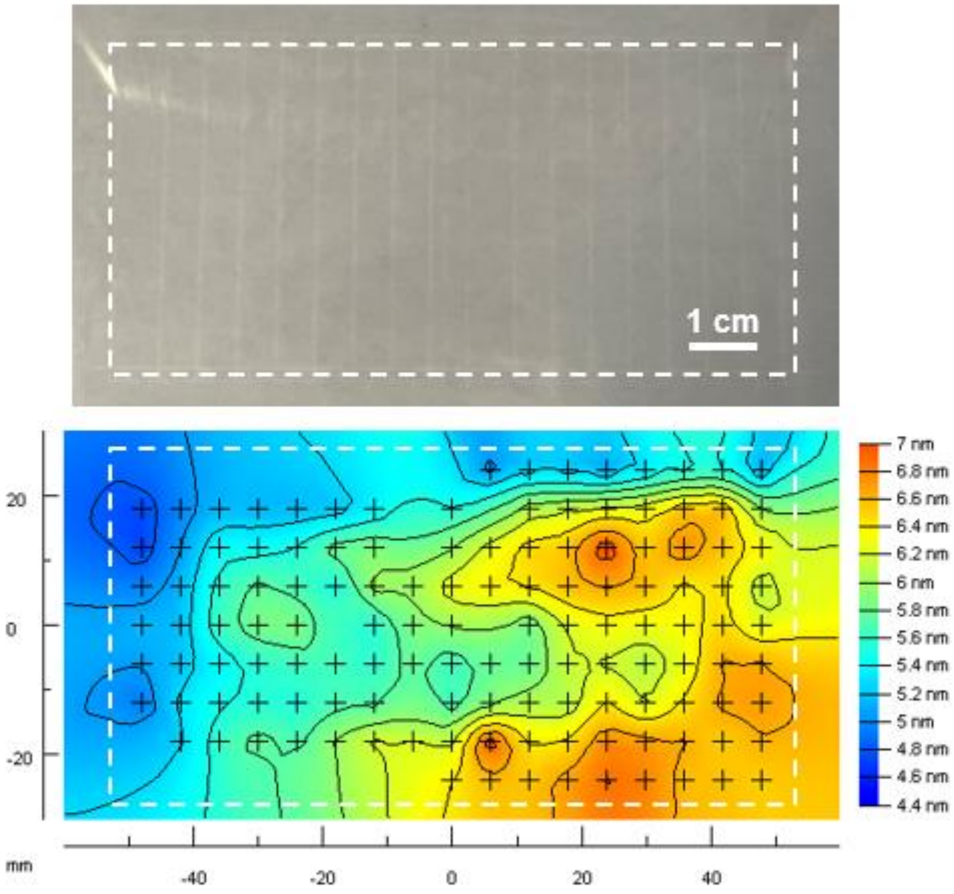

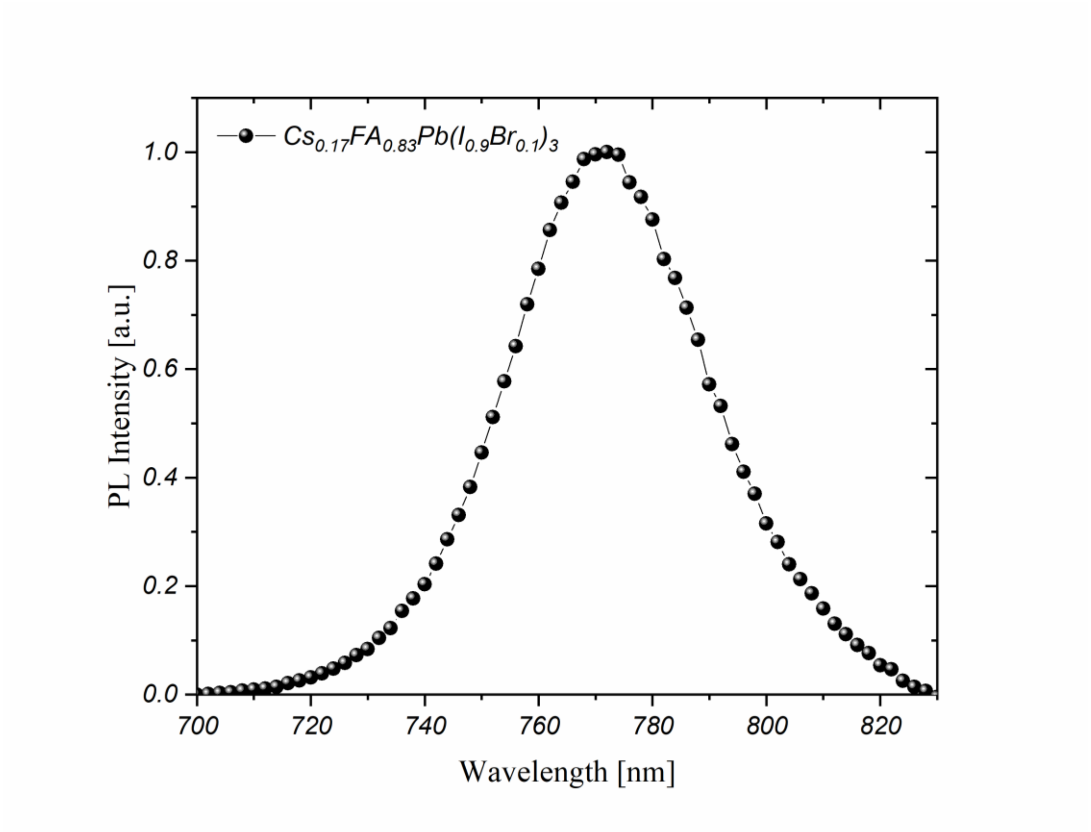

Figure S2. Photoluminescence spectra of $Cs_{0.17}FA_{0.83}Pb(I_{0.9}Br_{0.1})_3$ with peak at 772nm.

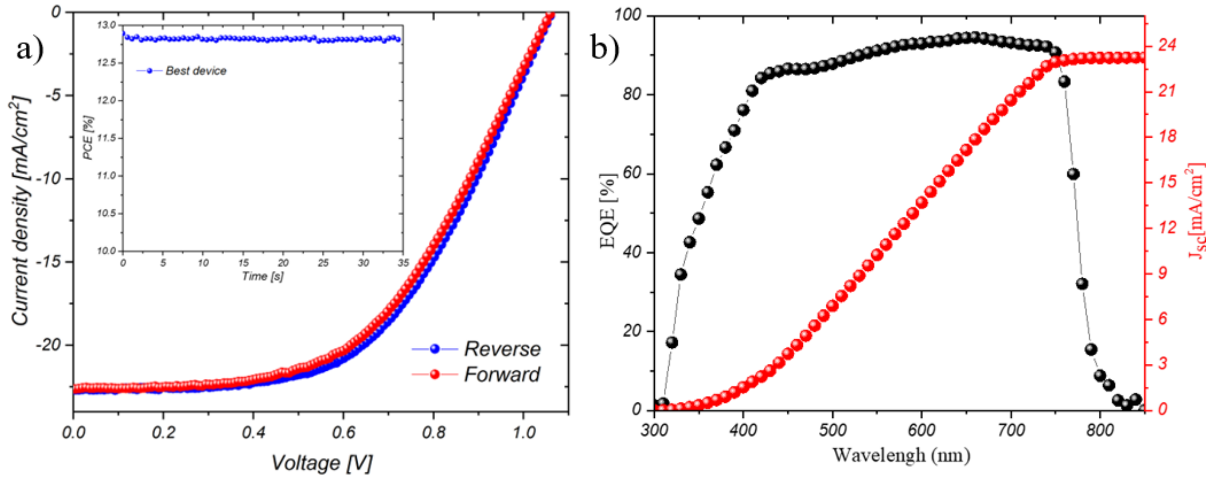

Figure S3. (a) J-V curve and stabilized efficiency of the best cell with active area of $0.616cm^2$. (b) EQE spectra of the best cell, showing an integrated J_{SC} of 22.75 mA/cm^2.

Table S2. Summary of the J-V results obtained on cell fabricated on best data.

Best	*Efficiency [%]*	*Fill Factor [%]*	*Voc [V]*	*Jsc [mA/cm2]*	*Integrated J_{sc} [mA/cm^2]*
Reverse	13.02	54.06	1.06	22.69	22.75
Forward	12.59	52.48	1.06	22.63	

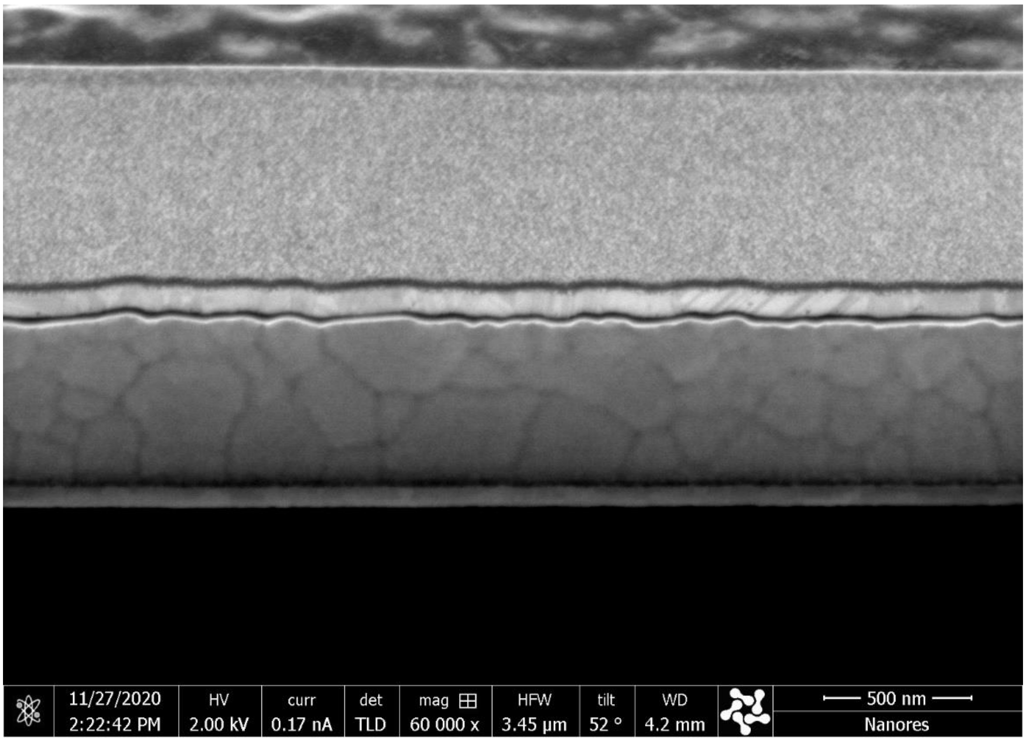

Figure S4. SEM cross section image of the device stack.

1. Matteocci, F.; Vesce, L.; Kosasih, F. U.; Castriotta, L. A.; Cacovich, S.; Palma, A. L.; Divitini, G.; Ducati, C.; Di Carlo, A., Fabrication and Morphological Characterization of High-Efficiency Blade-Coated Perovskite Solar Modules. *ACS Applied Materials & Interfaces* **2019,** *11* (28), 25195-25204.
